# Population assessment of health system performance in 16 countries

**DOI:** 10.2471/BLT.23.291184

**Published:** 2024-04-30

**Authors:** Margaret E Kruk, Shalom Sabwa, Todd P Lewis, Ifeyinwa Aniebo, Catherine Arsenault, Susanne Carai, Patricia J. Garcia, Ezequiel Garcia-Elorrio, Günther Fink, Munir Kassa, Sailesh Mohan, Mosa Moshabela, Juhwan Oh, Muhammad Ali Pate, Jacinta Nzinga

**Affiliations:** a Harvard TH Chan School of Public Health, 665 Huntington Ave, Boston, MA 02115, United States of America (USA).; b Ministry of Health and Social Welfare of Nigeria, Abuja, Nigeria.; c Milken Institute School of Public Health, George Washington University, Washington DC, USA.; d WHO Office on Quality of Care and Patient Safety, Athens, Greece.; e School of Public Health, Cayetano Heredia University, Lima, Peru.; f Institute for Clinical Effectiveness and Health Policy, Buenos Aires, Argentina.; g University of Basel and Swiss Tropical and Public Health Institute, Allschwil, Switzerland.; h Ministry of Health, Addis Ababa, Ethiopia.; i Public Health Foundation of India, New Delhi, India.; j College of Health Sciences, University of KwaZulu-Natal, Durban, South Africa.; k Seoul National University College of Medicine, Seoul, Republic of Korea.; l KEMRI-Wellcome Trust Research Programme, Nairobi, Kenya.

## Abstract

**Objective:**

To demonstrate how the new internationally comparable instrument, the People’s Voice Survey, can be used to contribute the perspective of the population in assessing health system performance in countries of all levels of income.

**Methods:**

We surveyed representative samples of populations in 16 low-, middle- and high-income countries on health-care utilization, experience and confidence during 2022–2023. We summarized and visualized data corresponding to the key domains of the World Health Organization universal health coverage framework for health system performance assessment. We examined correlation with per capita health spending by calculating Pearson coefficients, and within-country income-based inequities using the slope index of inequality.

**Findings:**

In the domain of care effectiveness, we found major gaps in health screenings and endorsement of public primary care. Only one in three respondents reported very good user experience during health visits, with lower proportions in low-income countries. Access to health care was rated highest of all domains; however, only half of the populations felt secure that they could access and afford high-quality care if they became ill. Populations rated the quality of private health systems higher than that of public health systems in most countries. Only half of respondents felt involved in decision-making (less in high-income countries). Within countries, we found statistically significant pro-rich inequalities across many indicators.

**Conclusion:**

Populations can provide vital information about the real-world function of health systems, complementing other system performance metrics. Population-wide surveys such as the People’s Voice Survey should become part of regular health system performance assessments.

## Résumé

**Objectif:**

Montrer comment l'enquête People's Voice Survey, un nouvel instrument comparable au niveau international, peut être utilisé pour nourrir la perspective d'une évaluation des performances du système de santé par la population dans des pays de tous les niveaux de revenus.

**Méthodes:**

Nous avons interrogé des échantillons représentatifs d'habitants de 16 pays à revenu faible, intermédiaire et élevé sur leur recours aux soins de santé, leur expérience en la matière et la confiance qu'ils y accordent sur la période 2022–2023. Nous avons ensuite résumé et visualisé les données correspondant aux principaux domaines du cadre de l'Organisation mondiale de la Santé relatif à la couverture sanitaire universelle pour évaluer les systèmes de santé. Enfin, nous avons examiné la corrélation avec les dépenses individuelles en soins de santé en calculant les coefficients Pearson, ainsi que les disparités de revenus au sein de chaque pays à l'aide de l'indice de pente de l'inégalité.

**Résultats:**

Dans le domaine de l'efficacité des soins, nous avons décelé des lacunes considérables au niveau des examens médicaux et de l'approbation envers l'offre publique de soins de santé primaires. Seul un répondant sur trois a fait état d'une excellente expérience en tant que patient lors des visites sanitaires, le pourcentage étant moins élevé dans les pays à revenu faible. Tous domaines confondus, c'est l'accès aux soins de santé qui a reçu la meilleure note; toutefois, à peine la moitié des personnes interrogées avaient la conviction de pouvoir bénéficier de soins de qualité à prix abordable si elles venaient à tomber malades. La qualité des systèmes de santé privés a été jugée supérieure à celle des systèmes de santé publics dans la plupart des pays. Enfin, la moitié seulement des répondants se sent impliquée dans la prise de décisions (voire moins dans les pays à revenu élevé). Par ailleurs, nous avons trouvé au sein de chaque pays des inégalités statistiquement significatives en faveur des catégories les plus aisées et ce, pour de nombreux indicateurs.

**Conclusion:**

Les populations peuvent fournir des informations vitales sur le fonctionnement réel des systèmes de santé, ce qui permet de compléter les autres mesures de performances. Les enquêtes telles que People's Voice Survey, menées auprès de l'ensemble des habitants, devraient désormais être intégrées aux évaluations périodiques des performances des systèmes de santés.

## Resumen

**Objetivo:**

Demostrar cómo el nuevo instrumento de comparación internacional, la *People's Voice Survey*, se puede utilizar para aportar la perspectiva de la población al evaluar el desempeño del sistema sanitario en países de todos los niveles de ingresos.

**Métodos:**

Se encuestaron muestras representativas de poblaciones de 16 países de ingresos bajos, medios y altos sobre la utilización de la atención sanitaria, la experiencia y la confianza durante 2022 y 2023. Se resumieron y visualizaron los datos correspondientes a los ámbitos clave del marco de cobertura sanitaria universal de la Organización Mundial de la Salud para la evaluación del desempeño de los sistemas sanitarios. Se examinó la correlación con el gasto sanitario per cápita calculando los coeficientes de Pearson y las desigualdades basadas en los ingresos dentro de los países utilizando el índice de desigualdad de la pendiente.

**Resultados:**

En el ámbito de la eficacia de la atención, se encontraron importantes deficiencias en los exámenes de salud y en la aprobación de la atención primaria pública. Solo uno de cada tres encuestados declaró tener una experiencia de usuario muy buena durante las visitas sanitarias, con porcentajes más bajos en los países de ingresos bajos. El acceso a la atención sanitaria fue el mejor valorado de todos los ámbitos; sin embargo, solo la mitad de las poblaciones se sentían seguras de poder acceder y costearse una atención de alta calidad en caso de enfermar. En la mayoría de los países, la población valoró más la calidad de los sistemas sanitarios privados que la de los públicos. Solo la mitad de los encuestados se sentían implicados en la toma de decisiones (menos en los países de ingresos altos). Dentro de los países, se encontraron desigualdades estadísticamente significativas a favor de los más ricos en muchos indicadores.

**Conclusión:**

Las poblaciones pueden proporcionar información vital sobre el funcionamiento real de los sistemas sanitarios, como complemento de otras métricas de desempeño del sistema. Las encuestas poblacionales, como la *People's Voice Survey*, deberían formar parte de las evaluaciones periódicas del funcionamiento de los sistemas sanitarios.

## ملخص

**الغرض:**

توضيح كيف يمكن استخدام الأداة الجديدة القابلة للمقارنة دولياً؛ People’s Voice Survey (مسح أصوات الناس)، للمساهمة بوجهة نظر السكان في تقييم أداء النظام الصحي في الدول على جميع مستويات الدخل.

**الطريقة:**

قمنا بمسح عينات تمثيلية من السكان في 16 من الدول ذات الدخل المنخفض والمتوسط والمرتفع، بشأن الاستفادة من الرعاية الصحية، والخبرة، والثقة خلال الفترة 2022 إلى 2023. قمنا بتلخيص وتصور البيانات المتعلقة بالمجالات الرئيسية لإطار التغطية الصحية الشاملة التابع لمنظمة الصحة العالمية، وذلك بغرض تقييم أداء النظام الصحي. قمنا بدراسة الارتباط مع نصيب الفرد من الإنفاق على الصحة من خلال حساب معاملات Pearson، وأشكال التفاوت القائم على الدخل داخل الدولة باستخدام مؤشر منحدر التفاوت.

**النتائج:**

في مجال فعالية الرعاية، وجدنا فجوات واسعة في الفحوصات الصحية، والمصادقة على الرعاية الأولية العامة. أبلغ واحد فقط من كل ثلاثة مشاركين عن تجربة مستخدم جيدة للغاية أثناء الزيارات الصحية، مع نسب أقل في الدول ذات الدخل المنخفض. تم تصنيف الحصول إلى الرعاية الصحية على أنه الأعلى بين كل المجالات؛ ومع ذلك، شعر نصف السكان فقط بالأمان بأنهم يستطيعون الحصول على رعاية عالية الجودة، وتحمل تكاليفها إذا أصيبوا بالمرض. قام السكان بتصنيف جودة الأنظمة الصحية الخاصة بأنها أعلى من جودة أنظمة الصحة العامة في معظم الدول. شعر نصف المشاركين فقط بالمشاركة في صنع القرار (أقل في الدول ذات الدخل المرتفع). وجدنا داخل الدول تفاوتات ذات دلالة إحصائية لصالح الأغنياء عبر العديد من المؤشرات.

**الاستنتاج:**

يمكن للسكان توفير معلومات حيوية حول وظيفة الأنظمة الصحية في العالم الحقيقي، مما يكمل مقاييس أداء النظام الأخرى. إن المسوح السكانية، مثل People’s Voice Survey، يجب أن تصبح جزءًا من التقييمات المنتظمة لأداء النظام الصحي.

## 摘要

**目的:**

展示如何利用具有国际可比性的新工具“大众之声调查”从民众角度评估不同收入水平的国家的卫生系统绩效。

**方法:**

在 2022-2023 年期间，我们从 16 个低收入、中等收入和高收入国家选取具有代表性的民众样本进行了调查，以了解其医疗保健服务使用情况和体验及其对此类服务的信任程度。我们基于世界卫生组织全民健康覆盖框架的各个关键领域进行了数据汇总和可视化处理，以用于卫生系统绩效评估。我们通过计算皮尔逊系数 (Pearson coefficient) 来衡量绩效与人均卫生支出的相关性，并使用不平等斜率指数来衡量各国国内收入的不平等程度。

**结果:**

在医疗保健有效性领域，我们发现在健康体检和对公共初级医疗保健服务的认可方面存在很大差距。只有三分之一的受访者表示，在就诊期间拥有非常好的就医体验，而低收入国家的这一比例更低。在所有领域中，对医疗保健服务可获得性的评分最高；然而，只有一半的受访者确信，他们生病时可以获得并负担得起高质量的保健服务。在大多数国家，人们对私营卫生系统的质量评分高于公共卫生系统。只有一半的受访者认为自己参与了相关决策（高收入国家的这一比例更低）。根据统计数据，我们发现这些国家的许多指标具有不平等性，即明显对富人更有利。

**结论:**

民众提供的卫生系统实际运行情况相关重要信息可有效补充说明其他系统绩效指标。应将全民调查（例如大众之声调查）作为定期进行的卫生系统绩效评估的一部分。

## Резюме

**Цель:**

Продемонстрировать, как новый, сопоставимый на международном уровне инструмент – опрос общественного мнения – может использоваться для учета мнения населения при оценке эффективности системы здравоохранения в странах с любым уровнем дохода.

**Методы:**

В период 2022–2023 гг. был проведен опрос репрезентативных выборок населения в 16 странах с низким, средним и высоким уровнем дохода по вопросам использования ресурсов здравоохранения, опыта и доверия к ним. Для оценки эффективности системы здравоохранения были обобщены и визуализированы данные, соответствующие ключевым областям всеобщего охвата населения услугами здравоохранения Всемирной организации здравоохранения. Кроме того, исследовалась корреляция с расходами на здравоохранение на душу населения путем расчета коэффициентов Пирсона, а также неравенство внутри страны по уровню дохода с помощью индекса ухудшения показателя неравенства.

**Результаты:**

В области эффективности оказания медицинской помощи были выявлены серьезные недостатки в проведении медицинских обследований и одобрении государственной первичной медицинской помощи. Только один из трех респондентов сообщил об очень хороших впечатлениях от посещения врача, причем в странах с низким уровнем дохода этот показатель ниже. Из всех областей доступ к медицинскому обслуживанию получил наивысшую оценку, однако только половина населения чувствовала уверенность в том, что в случае болезни сможет получить и оплатить высококачественное лечение. В большинстве стран качество частных систем здравоохранения оценивается населением выше, чем качество государственных систем здравоохранения. Только половина респондентов чувствует себя вовлеченной в процесс принятия решений (в странах с высоким уровнем дохода этот показатель ниже). Внутри стран наблюдается статистически значимое неравенство в пользу богатых по многим показателям.

**Вывод:**

Население может предоставить важную информацию о реальном функционировании систем здравоохранения, дополняя другие показатели эффективности системы. Опросы населения, такие как опрос общественного мнения, должны стать частью регулярных оценок эффективности системы здравоохранения.

## Introduction

The notion that health systems should be people-centred seems unexceptional. Health systems are occupied with serving people, are funded by people and aim to improve people’s health as their primary objective. However, concerns have grown in the past two decades that health systems have not matched the steadily rising expectations of patients, and are not delivering optimal outcomes or user experience.^1^^–^^7^ The global drive towards universal health coverage (UHC) also relies on a social compact that presumes the population finds health services to be of high value.

**Fig. 1:**
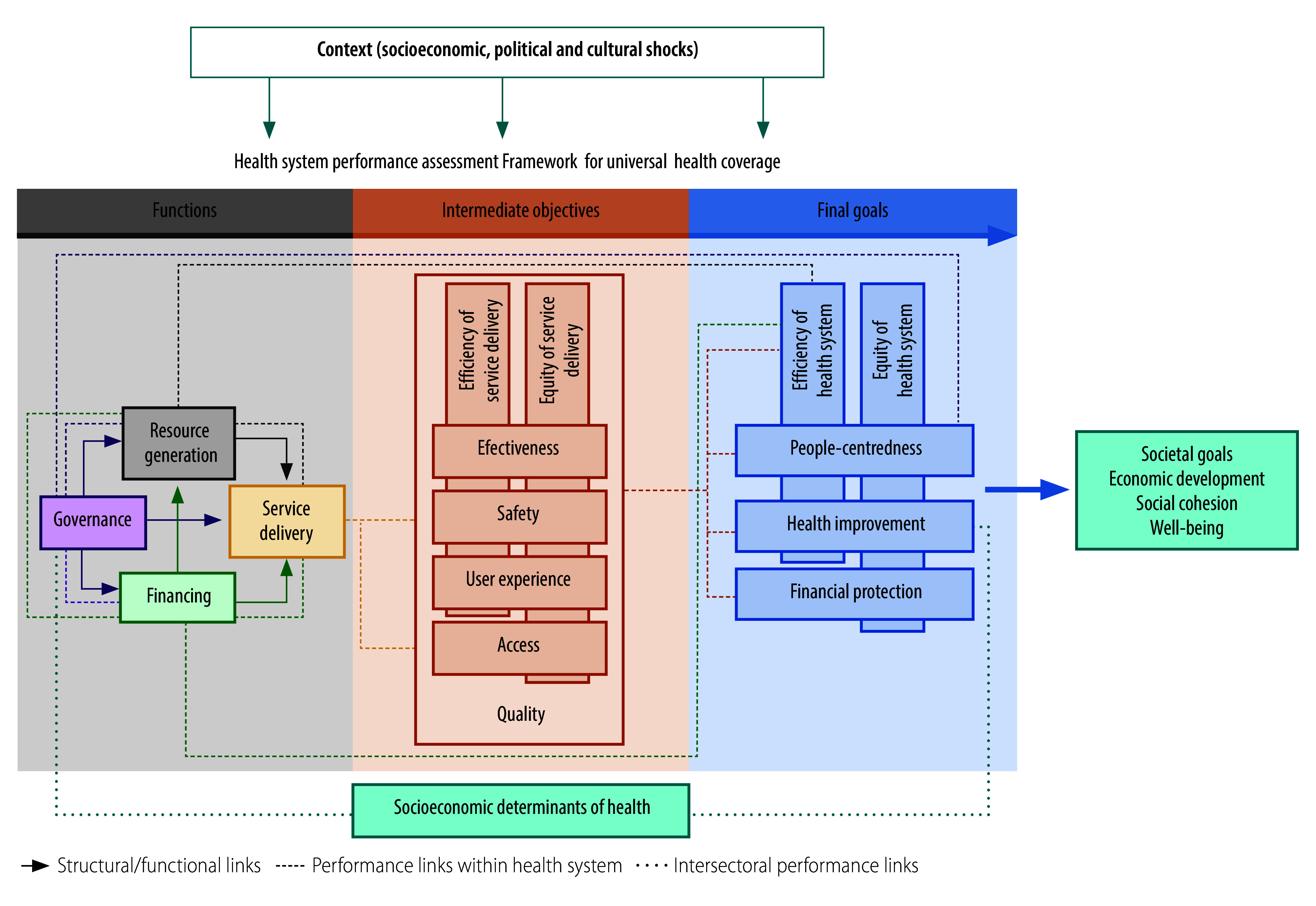
WHO framework used to define indicators in a study on the use of People’s Voice Survey indicators in a 16-country assessment of health system performance

Source: Adapted from World Health Organization, 2022.^11^

The integrated people-centred health services framework developed by the World Health Organization (WHO) calls for engaging communities and reorienting models of care to put people at the centre of health systems by expanding voice, co-production and choice.^8^^–^^10^ However, rhetoric on people-centredness has exceeded reality.^9^ The recently developed UHC framework for health system performance assessment (Fig. 1) highlights the need to evaluate health systems based on how they function for people and the outcomes they generate.^11^ Building on a body of literature that is arguing for a shift from measuring inputs to assessing health system function and health improvement,^12^^–^^14^ this emphasis on performance is especially relevant today with many health systems struggling in the aftermath of the coronavirus disease 2019 (COVID-19) pandemic. Incorporating people’s perspective in evaluating and steering health systems will require a robust and comparable set of measures obtained from the population.^15^

In this paper we describe the evaluation of health system performance by 16 different populations using the People’s Voice Survey (data publicly available in mid-2024),^16^ a new internationally comparable instrument.^17^ We hypothesize that the survey captures many of the domains in the WHO framework for health system performance assessment, and that it can provide unique insights into performance to complement other data sources. We report and discuss data from 16 countries on performance domains, and examine income-related inequality by analysing differences between income groups within each country. 

## Methods

The research presented here was undertaken by the Quality Evidence for Health System Transformation (QuEST) Network, a global research consortium on high-quality health systems.

### Data source

We acquired data via the People’s Voice Survey, a new instrument to measure experience and assessment of health system performance by people. The survey includes data describing demographics and health, utilization of care and system competence, care experience and quality, and confidence in a health system. The development and validation of the survey have been previously described.^17^ We adapted and translated the standard questionnaire for each health system context, and assessed our country-specific questionnaires for comprehension via cognitive interviews and/or pilot tests.

Research teams affiliated with the QuEST Network contracted survey research firms Ipsos and Social science research services (SSRS) to administer most of the surveys during May 2022 to July 2023. We obtained responses from population-representative samples of adults (age ≥ 18 years) in Argentina (Mendoza province only), Colombia, Ethiopia, Greece, India, Italy, Kenya, Lao People’s Democratic Republic, Mexico, Nigeria, Peru, Republic of Korea, South Africa, United Kingdom of Great Britain and Northern Ireland, United States of America and Uruguay. In most countries, we conducted surveys using computer-assisted telephone interviewing with a live interviewer. Respondents were sampled through random digit dial or known-list sampling. In Ethiopia and Kenya, where mobile phone penetration was less than 80%, we included supplemental face-to-face samples. In the Republic of Korea, the United Kingdom and the USA, we used nationally representative probability-based panels.

### Indicators

We based our survey indicators on the WHO UHC framework for health system performance assessment (Fig. 1),^11^ which has core areas of service delivery, intermediate objectives and final goals. The domains within these core areas are broadly consistent with many widely used frameworks.^11^^,^^14^^,^^18^^,^^19^ We identified survey data that corresponded to the concepts in the framework, and were able to quantify all intermediate objectives (except for safety) and all final goals (Fig. 1). We define the indicators used in our analysis in Box 1, mapping them to the framework constructs. Where possible, to better capture the core construct and to reduce statistical noise, we combined multiple variables from the survey.

Box 1 **WHO health system performance assessment framework domains and corresponding People’s Voice Survey indicators**

***Intermediate objectives***

- Care effectiveness: (i) public health effectiveness: percentage of respondents aged ≥ 40 years who had both a blood pressure and blood sugar test in the past year; (ii) quality of own care: percentage of respondents rating quality of care of most recent visit in past 12 months as very good or excellent; and (iii) quality of primary care services: average percentage of respondents rating three core primary care services (child, maternal, chronic disease) as very good or excellent.
- User experience: (i) respect: percentage of respondents rating respect that provider showed them and courtesy of office staff in most recent visit as very good or excellent, and who experienced no discrimination in health care; (ii) voice: percentage of respondents rating their desired level of involvement in their health care and their health-care provider’s explanation as very good or excellent; and (iii) customer service: percentage of respondents rating wait time and time spent with provider (as well as time waiting for appointment in six countries with appointment systems) as very good or excellent.
- Access: (i) connection to health system: percentage of respondents with usual source of care; (ii) use of needed health care: percentage of respondents with chronic disease who used care at least once in past year; and (iii) no unmet need: percentage of respondents with no unmet health care needs in past year.

***Final goals***

- People-centredness: (i) quality of public health system: percentage of respondents rating quality of the country’s public health system as very good or excellent; (ii) quality of private health system: percentage of respondents rating quality of the country’s private health system as very good or excellent; (iii) endorsement: percentage of respondents reporting that the health system works well as it is/needs only minor change; and (iv) involvement in decision-making: percentage of respondents rating that government considers public opinion as very good or excellent.
- Health improvement: (i) self-rated health: percentage of respondents reporting their overall health as very good or excellent; (ii) self-rated mental health: percentage of respondents reporting their mental health as very good or excellent; and (iii) absence of disease: percentage of respondents who do not have a chronic/longstanding condition.
- Financial protection: (i) insurance: percentage of respondents with any health insurance (public, private, other); and (ii) health security (affordability): percentage of respondents who are somewhat or very confident they can get and afford good care if they are sick.

In the intermediate objectives area of the framework, care effectiveness refers to the ability of the overall health-care system to provide essential and clinically effective services to those who need them.^19^ User experience refers to the provision of care that is respectful and aligns with individual preferences, needs and values.^20^ Access is the availability and timely delivery of health-care services.^21^

In the final goals area of the framework, people-centredness encompasses the ability of systems to capture the public’s input, perceptions of quality, choice of provider, engagement in care and trust in the system.^10^^,^^11^ Health improvement includes morbidity and mortality.^19^ For financial protection we included the weighted proportion of respondents with health insurance^22^ as well as perceived health security, developed as a people-reported measure of UHC.^23^

We also calculated domain score averages and plotted these against national health spending per capita; we used Excel (Microsoft, Redmond, USA) to calculate Pearson correlation coefficients (*r*).

### Statistical analysis

We constructed post-stratification weights according to country-specific demographic variables to account for differences in sample design and probability of selection. Numbers of respondents and percentages presented are therefore weighted. We captured demographic data to allow an equity analysis (online repository).^24^ We performed all analyses using Stata version 15.0 (StataCorp, College Station, USA). We created the circumplex plots (coxcombs) using R (R Core Team, Vienna, Austria) and the scatter plots using Excel.

To assess income-related inequalities within countries, we calculated the slope index of inequality (online repository).^25^ The slope index expresses the absolute percentage point difference in health system outcome between the predicted poorest and wealthiest in the income distribution, assuming a linear relation between income rank and the outcome.^26^ We used logistic regression and estimated the marginal effects using the lincom post-estimation command in Stata. We used within-country income group categories (online repository)^27^ to construct the equiplots.

### Ethics

The QuEST hub at Harvard, Boston, USA, and collaborators in participating countries obtained ethical clearance for the People’s Voice Survey as required by local regulations. As the survey presented minimum risk to participants, the Harvard Human Research Protection Program determined the research to be exempt from human subjects considerations. We provide details in the online repository.^28^

## Results

**Fig. 2:**
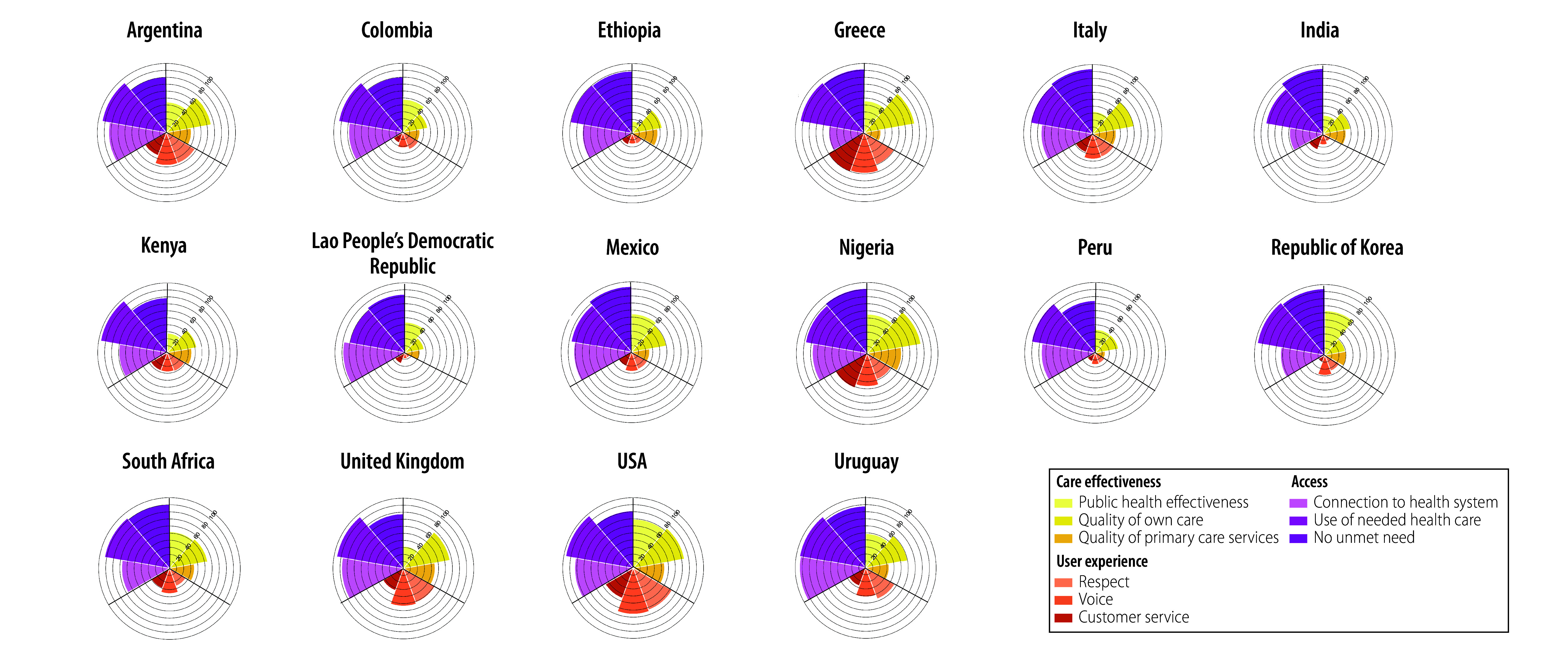
Survey responses to intermediate objectives indicators of health systems performance assessment: care effectiveness, user experience and access, 2022–2023

Note: See Box 1 for definitions of survey indicators.

**Fig. 3:**
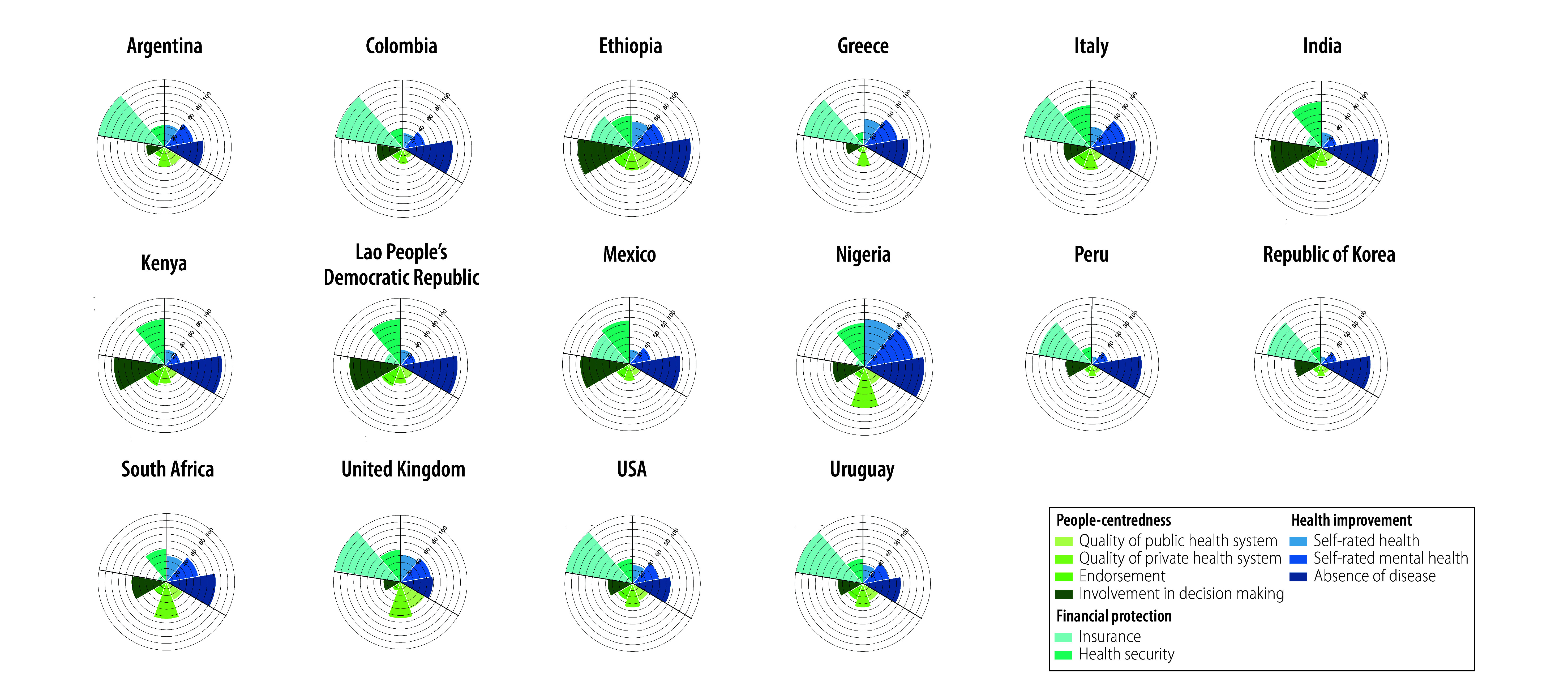
Survey responses to final goals indicators of health systems performance assessment: people-centredness, health improvement and financial protection, 2022–2023

Note: See Box 1 for definitions of survey indicators.

The number of participants in the 16 countries included in the survey totalled 27 795, ranging from 1001 (Italy) to 2779 (Ethiopia; Table 1 available at https://www.who.int/publications/journals/bulletin). We provide the weighted survey results for intermediate objectives and final goals in both data format (Table 1) and in coxcomb plots for visual interpretation (Fig. 2 and Fig. 3).

**Table 1:** People’s Voice Survey responses used to quantify domains of WHO framework for health system performance assessment, 2022–2023

Domain, indicator^a^	Weighted no. of respondents (weighted %)^a^																			
	Country, by World Bank income classification																			Total (*n* = 27 795)
	Low		Lower middle					Upper middle						High						
	Ethiopia (*n* = 2 779)		India (*n* = 2 004)	Kenya (*n* = 2 305)	Lao People’s Democratic Republic (*n* = 2 007)	Nigeria (*n* = 2 555)		Argentina (*n* = 1 190)	Colombia (*n* = 1 237)	Mexico (*n* = 1 002)	Peru (*n* = 1 255)	South Africa (*n* = 2 036)		Greece (*n* = 2 010)	Italy (*n* = 1 001)	Republic of Korea (*n* = 2 000)	United Kingdom (*n* = 1 677)	USA (*n* = 1 500)	Uruguay (*n* = 1 237)	
**Intermediate objective: care effectiveness**																				
Public health effectiveness**^b^**	164 (17.3)		246 (26.5)	219 (27.9)	423 (43.5)	510 (55.3)		347 (44.1)	300 (47.9)	279 (54.5)	213 (33.4)	467 (52.0)		588 (45.8)	235 (32.1)	860 (63.0)	341 (31.5)	623 (69.5)	352 (48.9)	6 166 (43.7)
Quality of own care**^c^**	729 (43.3)		426 (39.0)	762 (42.5)	356 (27.5)	1 444 (76.6)		677 (65.9)	373 (36.2)	392 (50)	319 (33.4)	830 (54.4)		1 247 (74.0)	484 (60.8)	557 (29.4)	969 (67.5)	1 019 (74.0)	650 (60.0)	11 232 (52.7)
Quality of primary care services**^d^**	79 (36.9)		651 (31.9)	804 (35.1)	423 (21.3)	1 229 (47.6)		190 (36.0)	1,237 (24.4)	255 (25.2)	179 (14.3)	713 (34.5)		478 (24.7)	337 (32.7)	610 (30.5)	755 (43.2)	666 (44.6)	406 (32.3)	9 200 (33.0)
**Intermediate objective: user experience**																				
Respect**^c^**	458 (16.5)		252 (12.6)	663 (28.8)	162 (8.1)	997 (39.0)		566 (47.6)	322 (26.0)	260 (25.9)	195 (15.5)	542 (26.6)		1 002 (49.9)	360 (35.9)	478 (23.9)	863 (51.5)	953 (63.5)	581 (47.0)	8 653 (31.1)
Voice	432 (15.6)		328 (16.4)	643 (27.9)	186 (9.3)	1 192 (46.6)		567 (47.6)	273 (22.0)	273 (27.2)	205 (16.4)	724 (35.5)		1 184 (59.0)	368 (36.8)	580 (29.0)	897 (53.5)	983 (65.6)	510 (41.3)	9 345 (33.6)
Customer service**^e^**	298 (17.7)		267 (24.9)	496 (27.7)	219 (16.8)	974 (51.6)		366 (35.6)	162 (15.8)	167 (21.3)	123 (12.9)	457 (29.9)		1 023 (60.7)	231 (29.0)	201 (10.6)	483 (33.4)	629 (45.7)	296 (27.3)	6 391 (30.0)
**Intermediate objective: access**																				
Connection to health system	1 991 (71.6)		973 (48.7)	1 598 (69.3)	1 740 (88.5)	1 976 (77.3)		993 (83.5)	967 (78.2)	820 (81.9)	956 (76.3)	1 366 (67.5)		1 035 (51.5)	746 (74.7)	1 258 (62.9)	448 (87.6)	1 244 (83.0)	1 154 (93.8)	20 265 (73.2)
Use of needed health care**^f^**	329 (90.1)		245 (84.1)	351 (97.1)	385 (80. 2)	279 (88.2)		463 (94.2)	314 (93.9)	204 (86.9)	286 (91.5)	514 (92.8)		622 (94.2)	294 (91.2)	762 (97.1)	807 (96.0)	594 (97.2)	508 (95.1)	6 955 (92.8)
No unmet need	2 474 (89.0)		1 877 (93.9)	1 811 (78.6)	1 669 (83.4)	2 336 (91.5)		955 (80.3)	990 (80.1)	933 (93.3)	931 (74.2)	1 843 (90.5)		1 846 (91.9)	938 (93.8)	1 881 (94.0)	1 279 (77.6)	1 217 (81.2)	1 087 (87.9)	24 067 (86.7)
**Final goal: people-centredness **																				
Quality of public health system	968 (34.9)		420 (21.9)	592 (25.8)	514 (25.6)	698 (27.4)		348 (29.7)	185 (15.0)	184 (18.9)	186 (14.9)	572 (28.1)		168 (8.6)	207 (20.9)	847 (42.4)	703 (42.3)	284 (18.9)	333 (27.3)	7 208 (26.2)
Quality of private health system	891 (33.1)		527 (27.6)	1 335 (59.0)	509 (26.1)	1 533 (60.2)		308 (30.2)	267 (22.5)	253 (25.7)	230 (18.5)	1088 (54.4)		572 (31.3)	318 (33.7)	651 (32.5)	672 (54.3)	616 (41.1)	426 (35.7)	10 197 (38.5)
Endorsement	829 (30.2)		622 (33.6)	582 (25.4)	744 (37.7)	458 (17.9)		199 (16.9)	229 (18.6)	227 (22.9)	180 (14.4)	434 (21.3)		219 (11.1)	311 (31.1)	821 (41.1)	239 (14.5)	344 (23.0)	321 (26.0)	6 758 (24.6)
Involvement in decision-making	2 180 (79.6)		1 440 (76.5)	1 409 (62.5)	1 553 (79.0)	1 192 (47.2)		316 (27.2)	476 (38.7)	730 (73.7)	495 (39.5)	1 047 (51.6)		500 (26.3)	395 (40.8)	1 058 (52.9)	427 (25.9)	535 (35.7)	439 (37.0)	14 192 (52.1)
**Final goal: health improvement**																				
Self-rated health	400 (33.7)		483 (24.1)	849 (36.9)	268 (13.3)	1800 (7 0.5)		400 (33.7)	287 (23.3)	223 (22.4)	154 (12.2)	779 (38.3)		815 (40.6)	319 (31.9)	490 (24.5)	692 (41.4)	683 (45.5)	346 (28.0)	9 689 (34.9)
Self-rated mental health	535 (45.0)		475 (23.7)	1 291 (56.1)	216 (10.8)	1 876 (73.4)		535 (45.0)	410 (33.2)	330 (32.9)	309 (24.7)	972 (47.8)		1 050 (52.3)	541 (54.0)	715 (35.8)	785 (46.9)	835 (55.8)	492 (39.9)	12 183 (43.9)
Absence of disease	696 (58.6)		1 710 (85.4)	1 944 (84.3)	1 526 (76.1)	2 235 (87.6)		696 (58.6)	901 (72.9)	766 (76.6)	942 (75.1)	1 481 (72.8)		1 348 (67.1)	677 (67.8)	1 215 (60.7)	811 (49.1)	889 (59.3)	346 (28.0)	20 257 (73.0)
**Final goal: financial protection**																				
Insurance	1 734 (62.4)		489 (24.6)	701 (30.5)	2 007 (100.0)	370 (14.5)		1 190 (100.0)	1 206 (99.4)	558 (58.8)	1 038 (82.9)	269 (13.3)		1 828 (91.1)	1 001 (100.0)	1 954 (99.1)	1 677 (100.0)	1 382 (92.2)	1 227 (100.0)	18 631 (67.4)
Health security	1 332 (48.3)		1 293 (69.2)	984 (43.0)	1 424 (71.3)	1 660 (65.0)		381 (32.9)	372 (30.7)	649 (65.8)	329 (26.4)	985 (48.5)		401 (21.0)	628 (63.9)	1 187 (59.4)	785 (48.8)	861 (57.7)	440 (37.1)	13 711 (50.3)

Note: The frequencies for number of respondents are rounded up to the nearest whole number as weighting can produce decimal frequencies.

^a^ See Box 1 for definitions of survey indicators.

^b ^The indicator only includes participants aged 40 years or older.

^c ^The indicator includes questions only asked to participants who reported a visit to a health facility in the last 12 months.

^d^ The indicator shows the average percentage of 3 questions. People were included if they had rated at least one of the questions as very good or excellent responses on the Likert scale.

^e ^The indicator includes an additional question about the time spent waiting for an appointment that was only asked in six countries with appointment systems (Greece, Italy, Mexico, Republic of Korea, United Kingdom and USA).

^f ^The indicator is only for participants with chronic disease.

### Intermediate objectives

We observed that the weighted proportion of people aged 40 years or older, who reported having had their blood pressure and blood sugar checked within the past year (public health effectiveness) was an average of 43.7% (6166/27 795) across all countries, with the highest weighted proportion in the USA (69.5%; 623/1500), followed by the Republic of Korea (63.0%; 860/2000), Nigeria (55.3%; 510/2555) and Mexico (54.5%; 279/1002). An average of 52.7% (11 232/27 795) rated their last visit as very good or excellent, from 27.5% (356/2007) in Lao People’s Democratic Republic to 76.6% (1444/2555) in Nigeria. Only 33.0% (4735/27 795) of respondents across all countries rated primary care services as high quality, with the highest weighted proportion in Nigeria (47.6%; 678/2555), followed by the United Kingdom (43.2%; 443/1677) and the USA (44.6%; 431/1500).

In the domain of user experience, the indicators respect, voice and customer service demonstrated similar ratings between countries; approximately one-third of respondents rated their last health-care visit as very good or excellent for these items. The highest endorsement of voice was observed in the USA (65.6%; 983/1500), followed by Greece (59.0%; 1184/2010), the United Kingdom (53.5%; 897/1677) and Argentina (47.6%; 567/1190). We noted the highest ratings for customer service in Greece (60.7%; 1023/2010), followed by Nigeria (51.6%; 974/2555) and the USA (45.7%; 629/1500). 

Measures for access received the highest endorsement of all the indicators in this study. The percentage of people with a usual source of care (i.e. connection to a health system) was 73.2% (20 265/27 795) across all countries, with the highest weighted proportion in Uruguay (93.8%; 1154/1237) followed by Lao People’s Democratic Republic (88.5%; 1740/2007) and the United Kingdom (87.6%; 1448/1677). We observed that use of needed health care among people with chronic illness was reported as greater than 80% in all countries. The percentage of people reporting no unmet need was also very high across all countries, and ranged from the lowest in Peru (74.2%; 931/1255) to the highest in the Republic of Korea (94.0%; 1881/2000; Table 1 and Fig. 2).

### Final goals

In the domain of health improvement, we observed that self-rated health and self-rated mental health received average ratings of 34.9% (9689/27 795) and 43.9% (12 183/27 795), respectively. The weighted proportion of respondents reporting an absence of disease was higher overall at an average of 73.0% (20 257/27 795) across countries, with the highest percentage in Nigeria (87.6%; 2235/2555), followed by Ethiopia (86.9%; 2414/2779), India (85.4%; 1710/2004) and Kenya (84.3%; 1944/2305).

With regards to people-centredness, the survey revealed that the quality of the public or government health system and the private health system was perceived as very good or excellent by only 26.2% 7208/27 795) and 38.5% (10 197/27 795) of respondents, respectively. We observed the highest percentage of people rating the government health system highly in the Republic of Korea (42.4%; 847/2000), closely followed by the United Kingdom (42.3%; 703/1677); in all other countries, around one third or less of respondents did not rate their country’s health system as being of good quality. Agreement that the health system is working well as it is (i.e. endorsement) received the lowest scores of all indicators in this analysis; we note an average value for this indicator of only 24.6% (6758/27 795). Overall, an average of 52.1% (14 192/27 795) of survey respondents agreed that their government considers the opinion of the public in health system decisions. This indicator was the most highly endorsed in Ethiopia (79.6%; 2180/2779), followed by Lao People’s Democratic Republic (79.0%; 1553/2007) and India (76.5%; 1440/2004).

Within the domain of financial protection, we note that an average of 67.4% (18 631/27 795) of respondents across all countries had insurance, with more than 90% of respondents in nine countries reporting to be covered by health insurance (Argentina, Colombia, Greece, Italy, Lao People’s Democratic Republic, Republic of Korea, United Kingdom, USA and Uruguay). The lowest level of insurance coverage was reported in South Africa (13.3%; 269/2036) and Nigeria (14.5%; 370/2555). Overall, an average of 50.3% (13 711/27 795) of people said that they could access and afford care if they were very sick (i.e. health security), with the highest scores for this indicator reported in Lao People’s Democratic Republic (71.3%; 1424/2007) and India (69.2%; 1293/2004; Table 1 and Fig. 3).

### Correlation with health spending

**Fig. 4:**
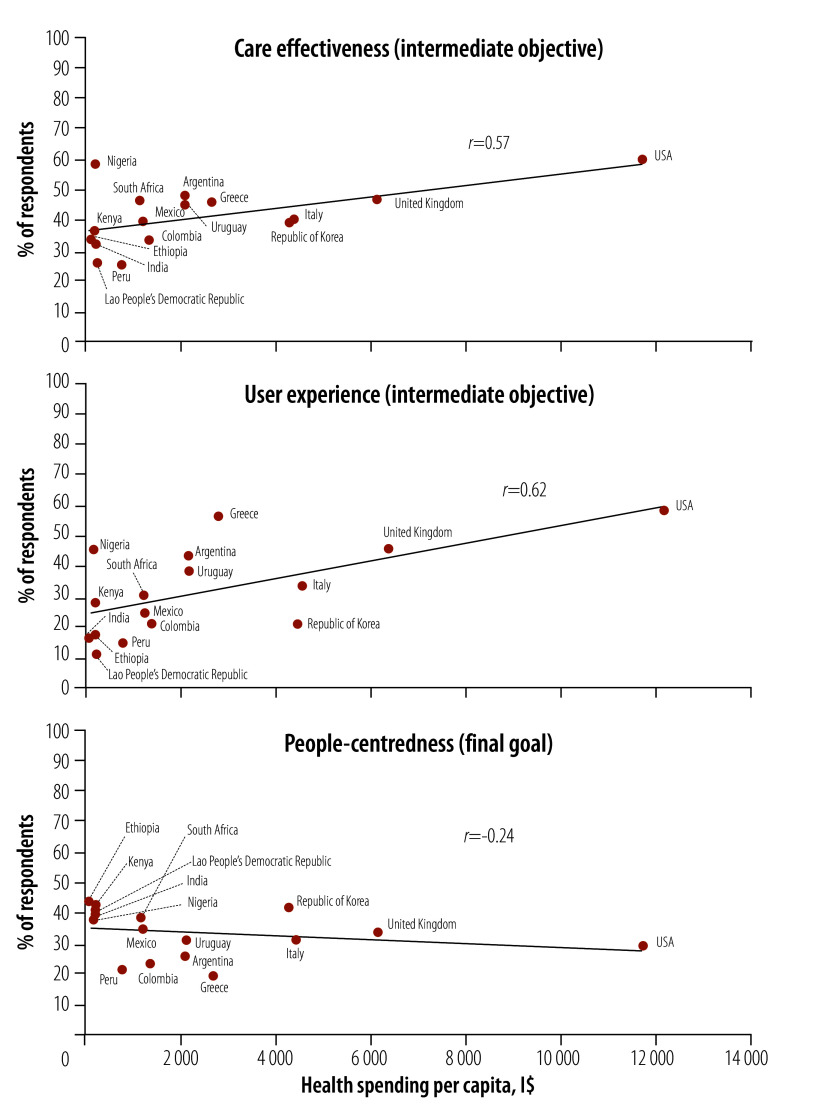
Correlation between average performance on care effectiveness, user experience and people-centredness and national health spending per capita, 2022–2023

We calculated how several of the performance indicators were correlated with national health spending per capita. We noted the strongest correlation with health spending for user experience (*r* = 0.62), followed by care effectiveness (*r* = 0.57), financial protection (*r* = 0.51) and access (*r* = 0.37; Fig. 4). We observed no association between total health expenditure per capita and health improvement (*r* = −0.03), and a negative correlation between spending and people-centredness (*r* = −0.24).

I$: international dollars.

Notes: Points indicate average performance across all indicators within each domain. See Box 1 for definitions of survey indicators.

### Income-based inequity

**Fig. 5:**
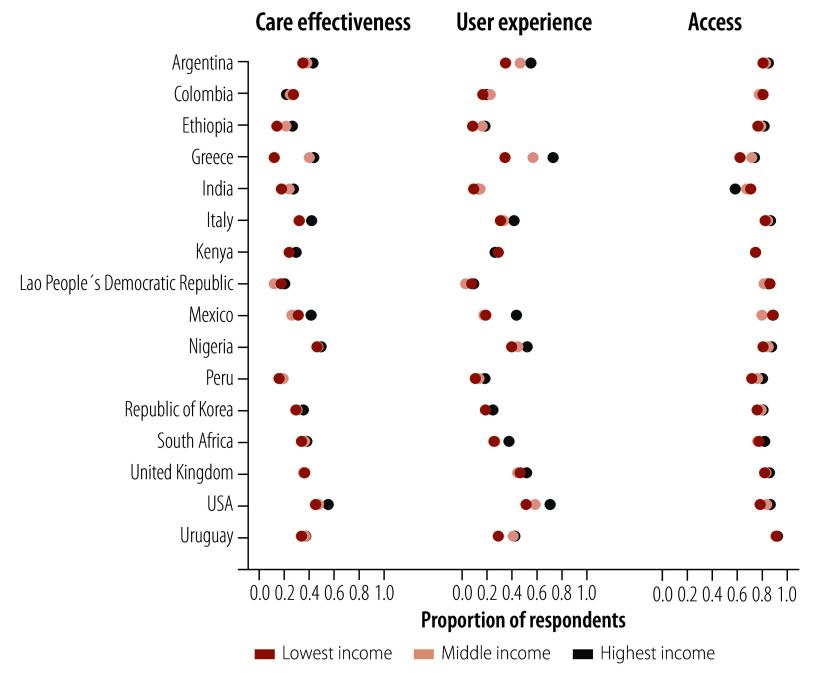
Ratings of intermediate objectives indicators by income category within each country, 2022–2023

Notes: Points indicate average performance across all indicators within each domain, in each income category. See Box 1 for definitions of survey indicators. Country-specific income categories are defined in online repository.^27^ Kenya includes the lowest and highest income groups only.

Fig. 5 shows that people with the lowest incomes within any specific country were less positive across the majority of the intermediate objective indicators. The results of final goals by income group are available in the online repository.^29^ We observe the greatest pro-rich inequities within Italy, Kenya, Lao People’s Democratic Republic, Mexico, Republic of Korea, South Africa and Uruguay in the domain of financial protection (online repository).^25^

Income-related inequities were also substantial within countries, with all countries except India and Peru showing a pro-rich difference of 25% or more between the richest and poorest respondents for at least one of the indicators (see slope index of inequality data in online repository).^25^ Our data highlight pro-poor differences for India for connection to health system (access) by 27%, and for Peru for endorsement (people-centredness) by 28%. The largest pro-rich inequalities were found for self-rated physical and mental health, and insurance. For example, compared with the lowest-income respondents, the weighted proportion of highest-income people reporting health insurance in Mexico was 54% higher, and the weighted proportion of highest-income people rating their health as very good or excellent in the United Kingdom was 47% higher (online repository).^25^

## Discussion

Despite efforts to improve the accountability of health systems to populations, assessments of health system performance have not prioritized people’s experiences and perspectives.^15^ Many of the aspects of performance obtained in the People’s Voice Survey are uniquely available from people, while other results provide complementary insights to other data sources. Data from the People’s Voice Survey highlighted several positive aspects of health system performance, but also uncovered major deficits.

Care effectiveness is a key signal of health system function. For example, fewer than half of respondents across countries (age ≥ 40 years) had received a blood pressure and blood glucose check in the past year. Screening and regular monitoring of these parameters in older adults is critical for early initiation of prevention and control measures for cardiovascular disease and diabetes, now the leading causes of disease, death and disability in most low- and middle-income countries.^30^^,^^31^ No country achieved over 50% positive ratings in the three core primary care services (maternal, child, chronic disease care). This result is consistent with objective evaluations of primary care quality in many settings.^32^^–^^36^ The low primary care ratings and the large divergence between people’s ratings of their own care and overall primary care may indicate a perception that good care is only available through the individual’s own efforts.^37^^,^^38^

Poor user experience can reduce care seeking and adherence, and undermine health outcomes and confidence.^39^^,^^40^ We found that respect, voice and customer service were rated positively by an average of only one in three respondents across countries studied. Ratings tended to be lower in lower income countries, and the correlation with national health spending was highest of all the domains. Historically, user experience has had more policy attention in wealthier countries.^41^ The findings here should provide motivation to policy-makers in all countries to pursue solutions, including via medical education, management, supervision and more responsive user feedback.

The domain of access demonstrated the best performance of all the intermediate objectives, although there is still work to be done to achieve universal coverage. In most countries, at least three quarters of people reported no unmet need for health care; similarly high proportions of people with chronic disease had at least one health-care visit in the past year. We observed greater variation in respondents reporting a usual source of care. Having a usual source of care is a (inexact) proxy for primary care, and is associated with a higher uptake of preventive services and a positive experience of care.^42^^,^^43^ However, although achieving high levels of access to care is important, high levels of effective coverage are required to improve health outcomes.^44^^–^^46^ The high levels of reported access in India, sub-Saharan African countries and parts of Latin America contrast with the substantial excess mortality from treatable conditions in these regions because of poor-quality care.^1^^,^^47^

In terms of people-centredness, feedback on the quality of public health systems was overall rather negative; only one in four respondents across the 16 countries rated their government health systems highly. In all countries, except Ethiopia and the Republic of Korea, private health systems outperformed public health systems. Health systems across the 16 countries are predominantly public, government-owned or based on social security. The exceptions are the Republic of Korea and the USA, where most health care is provided by the private sector. The gap between private and public health system approval serves as a measure of how far public services are lagging behind private services; if large, this gap can indicate a need for policy-makers to learn about what works in the private sector. On average, only half of respondents felt their governments considered their opinions when making health policies. Other studies found that users in both low- and high-income countries are dissatisfied with their health systems.^14^^,^^48^

Our observation that people-centredness measures are negatively correlated with health spending is a result of lower endorsement and poor government responsiveness to user feedback in wealthier countries. This finding requires further study, but suggests that spending on health does not prevent populations from feeling alienated from their health systems. One implication is that policy-makers should more meaningfully involve the population in system reform, and should increase their efforts to better communicate the work and achievements of the health system to the population.^49^

The health improvement measures in the People’s Voice Survey (self-rated health, self-rated mental health and no chronic disease) reflect social determinants of health and public health, as well as health systems. These indicators are therefore not as well-suited to measuring health system performance as other more specific health system metrics, such as amenable mortality.^1^^,^^2^^,^^50^

With regards to financial protection, we observe that insurance does not buy health security. Although an average of two thirds of the respondents had health insurance, only half of the respondents felt they could access and afford good-quality care if very sick. This perceived poor health security could be considered a measure of the effectiveness of UHC.^23^ Although financial protection received higher ratings in wealthier countries, there were still major shortcomings. In the nine countries with universal health insurance (> 90% coverage), less than half of respondents felt health-secure. These findings are consistent with data on high out-of-pocket spending in countries with universal or near-universal health insurance, and with a recent rise in the purchase of private insurance in some high-income countries.^51^^–^^53^ These findings have important implications for the design of health benefit packages, and for timely and effective delivery of insured services.

We observed that perceived and/or experienced health system performance varied by respondents’ incomes. Although access ratings were consistent between the various income groups within most countries, we found poorer ratings of care effectiveness among lower-income respondents in four countries. We noted the largest inequities in user experience, with around one half of the countries showing a pro-rich pattern. Similar wealth inequities in respectful treatment have been found in other studies.^54^^–^^56^ These data may underestimate inequities as poorer and less educated respondents may have lower expectations and positively rate low-quality care.^57^

Our study had several limitations. First, the People’s Voice Survey was developed using the conceptual framework of the *Lancet* *Global Health* Commission on High Quality Health Systems and did not contain all indicators within the WHO framework (e.g. safety).^17^ Second, people’s perceptions of health systems are influenced by various cultural, political, social and personal factors, including education level, as well as individual health and the effect of any health care received.^58^ Because the recent COVID-19 pandemic may have influenced people’s responses, the survey should be repeated every 2 years to gain an understanding of performance and trajectory. Third, in some countries the prevailing low quality of health care may reduce people’s expectations and therefore inflate ratings, complicating cross-country comparison. Comparisons are therefore most valid for countries with similar income levels and health systems.^57^ Fourth, the survey sampled the entire population and did not have sufficient resolution to reflect the experiences of potentially vulnerable subgroups (e.g. recent migrants, homeless or older populations), who may have very different perceptions and experiences of a health system; to begin to address this limitation, QuEST collaborators are currently pursuing studies focused on migrants and adolescents.

We found that the elements of the WHO framework were relevant and measurable (except for safety) from the survey responses. One area that could be further refined in the framework is people-centredness. Since this element is at the core of the health system, the concept might be integrated throughout the other dimensions of the framework (e.g. voice as part of user experience). Further, given the central role of trust in the health system, confidence (including health system endorsement and health security) could be added as an impact indicator of system performance.^23^

To conclude, we have shown that populations can provide rich and nuanced information about the function of their health systems. These data not only describe health system performance but also provide signals of strength and weakness to guide policy. We found that in all countries people are generally unsatisfied with their current health system, suggesting that major reforms, particularly those that are co-designed with users, may find a receptive public.^59^ Reforms are particularly urgent given the waning confidence in public health systems at a time when people’s aspirations for good health care have never been higher. 
